# Comparison of Phototactic Behavior between Two Migratory Pests, *Helicoverpa armigera* and *Spodoptera frugiperda*

**DOI:** 10.3390/insects13100917

**Published:** 2022-10-09

**Authors:** Yong Wang, Yajun Chang, Sai Zhang, Xingchuan Jiang, Bin Yang, Guirong Wang

**Affiliations:** 1 State Key Laboratory for Biology of Plant Diseases and Insect Pests, Institute of Plant Protection, Chinese Academy of Agricultural Sciences, Beijing 100193, China; 2 Hubei Key Laboratory of Quality Control of Characteristic Fruits and Vegetables, Hubei Engineering University, Xiaogan 432000, China; 3 College of Plant Protection, Anhui Agricultural University, Hefei 230036, China

**Keywords:** *Spodoptera frugiperda*, *Helicoverpa armigera*, light trap, phototactic rate

## Abstract

**Simple Summary:**

The light trap is a pesticide-free method for pest control. Appropriate wavelength and light intensity are the key factors for trapping specific target pests. However, present light-trapping methods mainly use UV light, which is effective with *Helicoverpa armigera* and many nocturnal insects and has displayed a low effect on an important migratory pest, the fall armyworm, *Spodoptera frugiperda* (Lepidoptera: Noctuidae). In this study, a series of phototactic behavioral assays were carried out and physical parameters were included to identify the different phototactic behaviors between *S. frugiperda* and *H. armigera*. It was found that *S. frugiperda* had the highest average phototactic rate to blue light than other lights. The phototactic rates of the two moths increased gradually with light intensity and were not obviously influenced by sex. Meanwhile, phototactic rates of *S. frugiperda* were significantly lower than those of *H. armigera* at a low light intensity of UV light. Combined with these results and the obtained formula, we summarized a proposal of using blue light for light traps to control *S. frugiperda*. These results provide an experimental and theoretical basis for improving light-trapping techniques for managing *S. frugiperda*.

**Abstract:**

The fall armyworm, *Spodoptera frugiperda* (Lepidoptera: Noctuidae), is an important migratory pest, causing great losses to agricultural production. Light trapping is a pesticide-free method for pest control and is influenced by many factors, especially wavelength and light intensity. In this study, a series of phototactic behavioral assays were carried out and the physical parameters were included to identify phototactic responses of *S. frugiperda*, with *Helicoverpa armigera* as control. It was found that *S. frugiperda* showed the highest average phototactic rate to blue light among five different LED lights. The phototactic rates of the two moths increased gradually with light intensity and were not obviously influenced by sex. In addition, the phototactic rate of *S. frugiperda* was significantly lower under a low light intensity of UV light than that of *H. armigera*, further confirmed by the indoor simulation experiment and EC50. According to the obtained parameters, the trapping distance of *S. frugiperda* to blue light was smaller than that of *H. armigera* to UV light. Therefore, we summarized a proposal of using blue light for light traps to control *S. frugiperda*, with a maximum distance of no more than 108 m. These results provide an experimental and theoretical basis for improving light-trapping techniques for managing *S. frugiperda*.

## 1. Introduction

The fall armyworm, *Spodoptera frugiperda* (Lepidoptera: Noctuidae), is native to tropical and sub-tropical areas of the Americas and spread to Mexico, the United States, and Canada in the mid-19th century [1]. As an invasive alien species, *S. frugiperda* was first discovered in Nigeria and Ghana, Africa in early 2016 and caused USD 13 billion in economic losses in sub-Saharan Africa [2]. In the past four years, it has invaded 47 African countries, 18 Asian countries, and now Australia has also been reported [3]. Because of its harmfulness and invasiveness, it was rated as one of the top ten pests in 2017 (Wild 2017) [4,5]. At least four characteristics are conducive to its invasion and harm, including a wide range of host plants, high fecundity, strong migration, and the rapid development of resistance to a range of insecticides/viruses [6,7,8,9,10]. Various control technologies have been applied to the control of *S. frugiperda*. Due to its phototaxis, light-trap technology is one of the methods of IPM (Integrated Pest Management). However, the phototactic behaviors and mechanisms are still unclear, and there are relatively few studies [5].

Due to various living habits and biological and ecological characteristics, the phototaxis of insects to light sources is diversified among species [11,12]. UV light or light sources with high UV content are usually more attractive to insects [13]. For example, UV LED was more useful for trapping the Angoumois grain moth, *Sitotroga cerealella*, among the six light sources [14]. However, many moths were more sensitive to blue/green lights or other lights. Three moths, *Mythimna separata*, *S. exigua*, and *S. litura*, were sensitive to green light [15,16]. The Indian meal moth, *Plodia interpunctella*, has a significantly stronger attraction to violet light than to UV light at maximum light intensity, and in certain conditions, the attraction to violet light was not affected by UV light [17]. Later, the relative attractiveness of 19 monochromatic LED lights to *Helicoverpa armigera* was tested in fields, and the results showed that the highest attraction was obtained with UV, but it was not significantly different from purple light [18]. Clearly, the phototactic behaviors toward light sources vary among nocturnal moths [19]. Besides, even for the same insect, the phototactic rate varied with light intensity after being exposed to the same light wavelength [20,21]. Four relationships between phototactic rate and light intensity, i.e., positive correlation, negative correlation, wavy correlation, and uncorrelation were discovered in different insects [22,23,24,25]. Therefore, it is necessary to study the species-specificity of phototactic behavior, light source, and light intensity in different species [20,26].

Both *S. frugiperda* and *H. armigera* are migratory pests of Noctuidae, with certain phototaxis; however, field experiments revealed that black lights for commercial applications trapped these two moths differently. It had proven to be effective on *H. armigera* but not for *S. frugiperda*, since only a few or even individual moths were caught in the wild [27]. At the same time, the trapping amount of male and female insects by conventional light traps was different. The results of different teams and different field experiments were not consistent, and all three situations existed in the ratio of male to female, i.e., >1, <1, and equal to 1 [27,28,29]. It is not clear whether this difference is caused by the difference in phototaxis between males and females or the difference in spatial distribution between males and females. Because they both belong to noctuid pests and have phototaxis, it would be an interesting topic to compare their phototactic differences and phototactic mechanisms.

Phototactic responses of insects are influenced by many factors, especially the nature of the light source (wavelength and light intensity) [22,30]. In this study, a series of laboratory experiments were conducted to understand the effects of spectral sensitivity and light intensity on the phototactic behavior of *S. frugiperda* and *H. armigera.* In addition, the phototactic reactions between males and females were compared. The results showed that *S. frugiperda* had the highest average phototactic rate to blue light than other LED lights, compared to *H. armigera*, which showed the maximal attraction to UV light. These were confirmed by phototactic recovery experiments. The phototactic rate was increased with light intensity and was not influenced by sex. In addition, we provided the trapping distance as guidance for controlling *S. frugiperda* by light traps. These findings offer an experimental and theoretical foundation for enhancing the light-trapping technique for *S. frugiperda* management.

## 2. Materials and Methods

### 2.1. Insects

*S. frugiperda*, captured in the Anhui Province of China in 2020, was supplied by the Anhui Agricultural University. *H. armigera*, captured in the Hebei Province of China in 2020, was raised in our laboratory. Larvae of *S. frugiperda* were fed on an artificial diet purchased from Mitaka Boeki Co., Ltd. (Tokyo, Japan), while the larvae of *H. armigera* were fed on a widely used artificial diet. Males and females were identified according to the abdominal end of the pupae and placed in different cages (20 × 20 × 30 cm) providing 10% honey water. Insects were maintained at 25 ± 1 °C, 70 ± 10% RH with a photoperiod of L14:D10 in the insect chamber or the insectarium. Two-day-old adults were used for the experiments.

### 2.2. LED Light Sources and Intensity Design

The wavelength required for the experiment was customized by the company. The descriptions of LED lights and light intensities were adopted according to the literature (Table S1). Because most moths had a trichromatic vision system, UV (365 ± 5 nm), blue (420 ± 10 nm), and green (520 ± 10 nm) LEDs were selected. Meanwhile, as the moths were not sensitive to red light as previously reported [31], red (620 ± 10 nm) and white LEDs (6205 K) were used as controls. The power range of the light trap used in the field was 8–20 watts, and an intermediate value of 15W LEDs was adopted for the behavior test. Based on the light intensity in other experiments [5] and the light intensity of our insect room, the following five light intensities were selected, about 2000, 200, 50, 5, and 0.5 lx. An LED light was placed on the side of the lamp-lit cage away from the plastic pipe. In addition, the light intensity was measured directly below the LED. All LED lights were purchased from Shenzhen Yijiaxin Electronics Co., Ltd. (China), and the light intensities were measured by a digital lux meter (TA8126B, Suzhou Tiansi Electronic Industry Co., Ltd., China). The spectrum of LEDs used for the experiments is shown in Figure S1.

### 2.3. Phototactic Behavior Tests

The phototactic responses of the moths were tested in a chamber within a dark room, similar to the experimental device designed by others [5]. In brief, the behavior reaction box was composed of two white insect cages (40 cm × 40 cm × 40 cm), which were wrapped in opaque black cloth during the experiments and connected through a black plastic pipe channel (25 cm in diameter and 200 cm in length). In order to simulate the field environment, the adults were first allowed into the darkness for half an hour before the LED light was turned on, and the number of moths in the light area was counted after the dark period ended. As a control, the number of adults in the light area without light was counted after the dark period. The phototactic behavior responses of male and female moths were checked individually and more than sixty individuals were used at least three times, then the number of females and males in the light area was recorded separately. Phototactic rate (%) = (number of moths in the light area/total number of moths) × 100; relative phototactic rate = experimental phototactic rate - control phototactic rate.

### 2.4. Phototactic Recoveries of Two Moths under Indoor Simulated Conditions

The experiment was performed in a dark room (4 m × 2 m × 3 m). At one end of the dark room, three LED lights (UV, blue, and green) with 0.5 lx were used and an opaque plastic basin with a diameter of 20 cm was placed containing a certain amount of detergent solution. On the other end, male moths were released after half an hour of dark adaptation. The number of moths in the basin was counted the next day. The temperature and humidity conditions were consistent with the adult feeding conditions. Each treatment was repeated for at least three repetitions with 20–30 moths each time.

### 2.5. EC50 and Effective Distances of Two Moths to Different Wavelength Light Sources

In order to further evaluate the attraction effect of each light source, EC50 (light intensity causing a half-maximal phototactic response) of different light sources was calculated to compare the light intensity when reaching the same attractive effect. Previous results showed there was no significant difference in the phototactic rates of both sexes to different light sources; therefore, the phototactic rates of both sexes were combined and not compared separately. The fitting equation was obtained using the software GraphPad Prism version: 9.3.0, and the light intensities were converted by log. EC50 and refitted by the nonlinear regression curve fit and log (agonist) vs. the normalized response-variable slope. The Hill equation was: P = Bottom + (Top– Bottom)/(1 + 10^((Log EC50 – I) × Hill slope)). P was the phototactic rate caused by light intensity and I was the logarithm of light intensity. The relation function between light intensity and distance was: I = X/D^2^. I was the light intensity and D was the distance. X meant the luminosity and could be estimated by detecting the illuminance at different distances from the light source. Combining the two equations, the distance could be calculated under the given phototactic rate and light intensity.

### 2.6. Statistical Analysis

Data were presented as the mean ± SE. Student’s t-test was used for the comparison of pairs, and ANOVA was used for different groups followed by Duncan’s multiple comparisons for the determination of significant differences (*p* < 0.05). Statistical analyses were performed with IBM SPSS Statistics 25 software (SPSS Inc., Chicago, IL, USA).

## 3. Results

### 3.1. Phototactic Rates of Two Moths to Different Wavelength Light Sources

**Figure 1:**
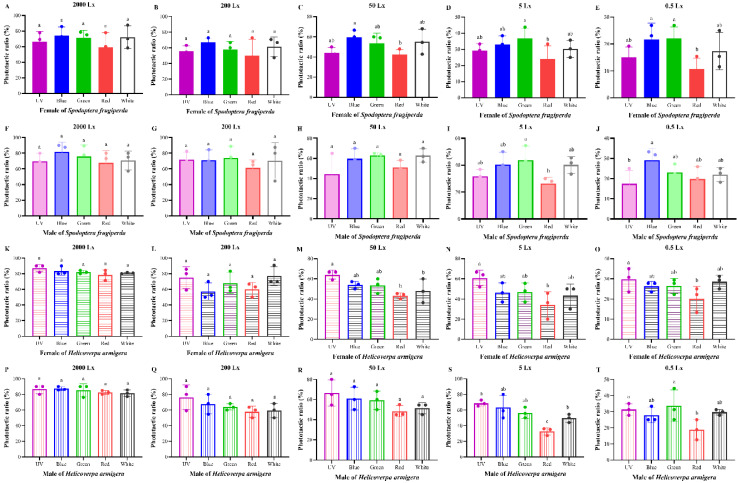
Phototactic rates of *S. frugiperda* and *H. armigera* to different wavelength lights. Data are mean ± SE. (**A**–**E**), phototactic ratios of female *S. frugiperda* to five lights under different light intensities ((**A**) for 2000 lx; (**B**) for 200 lx; (**C**) for 50 lx; (**D**) for 5 lx; (**E**) for 0.5 lx). (**F**–**J**), phototactic ratios of male *S. frugiperda* to five lights under different light intensities ((**F**) for 2000 lx; (**G**) for 200 lx; (**H**) for 50 lx; (**I**) for 5 lx; (**J**) for 0.5 lx). (**K**–**O**), phototactic ratios of female *H. armigera* to five lights under different light intensities ((**K**) for 2000 lx; (**L**) for 200 lx; (**M**) for 50 lx; (**N**) for 5 lx; (**O**) for 0.5 lx). (**P**–**T**), phototactic ratios of male *H. armigera* to five lights under different light intensities ((**P**) for 2000 lx; (**Q**) for 200 lx; (**R**) for 50 lx; (**S**) for 5 lx; (**T**) for 0.5 lx). Means with different letters are significantly different according to Dunnett’s test (*p* < 0.05, ANOVA). UV, UV LED; Blue, blue LED; Green, green LED; Red, red LED; White, white LED.

As a negative control, the phototactic rate in the light area without light was calculated after the dark period (Figure S2). Five light sources (365 nm, 420 nm, 520 nm, 620 nm, and white) were tested for the attractiveness of *S. frugiperda* and *H. armigera* (Figure 1). The two pest species displayed a certain phototactic response to all light sources. On the whole, the difference in phototactic rate between different light sources was not obvious in our experiments.

For females of *S. frugiperda*, there were no significant differences between the five light sources at high light intensity (2000 and 200 lx). When the light intensity was at the medium light intensity of 50 lx, the attraction rate of blue light was observably higher than that of red light (*p* < 0.05). Nevertheless, the phototactic rate of females to green light was remarkably higher than that of red light when at a low light intensity of 5 lx (*p* < 0.05). Additionally, male moths had a higher phototactic rate to green light than that of red light at 5 lx and a significant difference in the phototactic rate was recorded between blue and UV light (*p* < 0.05).

For *H. armigera*, the phototactic rates of females to UV light were higher than that of red light at the medium- and low-light intensities (50, 5, and 0.5 lx, *p* < 0.05). Similarly, there were significant differences in the phototactic rates between UV light and red light at low light intensities of 5 and 0.5 lx (*p* < 0.05). Red light had the lowest phototactic rate for both moths. Overall, *S. frugiperda* was sensitive to blue and green light, while *H. armigera* was sensitive to ultraviolet light.

**Figure 2:**
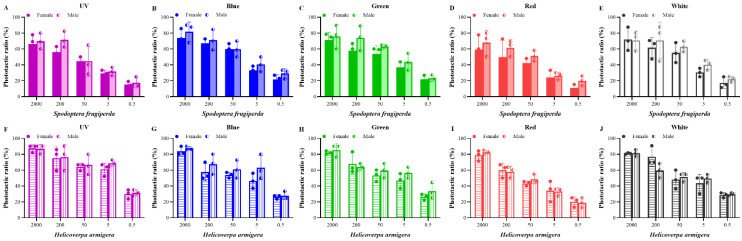
Phototactic rates of male and female adults of *S. frugiperda* and *H. armigera*. (**A**–**E**), phototactic ratios of male and female adults of *S. frugiperda* to different wavelength lights under five light intensities ((**A**) for UV light; (**B**) for blue light; (**C**) for green light; (**D**) for red light; (**E**) for white light). (**F**–**J**), phototactic ratios of male and female adults of *H. armigera* to different wavelength lights under five light intensities ((**F**) for UV light; (**G**) for blue light; (**H**) for green light; (**I**) for red light; (**J**) for white light). Data are mean ± SE. Means between the two groups under the same light intensity were compared according to Student’s *t*-test (*p* < 0.05). UV, UV LED; Blue, blue LED; Green, green LED; Red, red LED; White, white LED.

In addition, there were no obvious differences in the phototactic rates between the sexes of the two moths to the same light source and light intensity, though the phototactic rates of males were slightly higher than those of females (Figure 2); therefore, the phototactic rates of male and female moths were combined and were not compared separately later.

### 3.2. Phototactic Rates of Two Moths to Different Light Intensities

**Figure 3:**
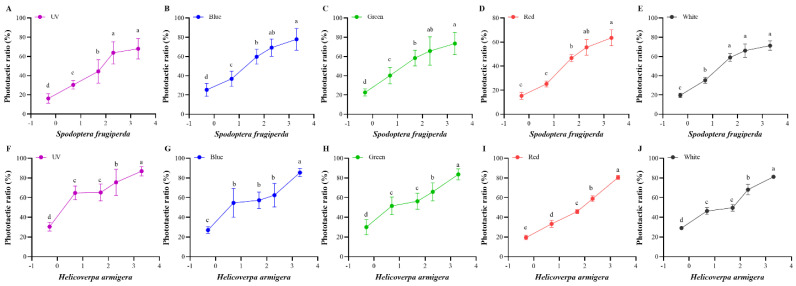
Phototactic rates of *S. frugiperda* and *H. armigera* to different intensities. (**A**–**E**), phototactic ratios of *S. frugiperda* to different wavelength lights under five light intensities ((**A**) for UV light; (**B**) for blue light; (**C**) for green light; (**D**) for red light; (**E**) for white light). (**F**–**J**), phototactic ratios of *H. armigera* to different wavelength lights under five light intensities ((**F**) for UV light; (**G**) for blue light; (**H**) for green light; (**I**) for red light; (**J**) for white light). The light intensity is 0.5, 5, 50, 200, and 2000 lx, and is transformed into log10 values. Data are mean ± SE. Means with different letters are significantly different according to Dunnett’s test (*p* < 0.05, ANOVA). UV, UV LED; Blue, blue LED; Green, green LED; Red, red LED; Black, white LED.

The effects of light intensity on the attraction for the two moths were tested (Figure 3). For *S. frugiperda*, the phototactic rate increased with the increase in light intensity, showing an intensity-dependent manner. However, this increase was regardless of the wavelength detected. There were obvious differences between the low (0.5 and 5 lx) and high (200 and 2000 lx) light intensities (*p* < 0.05). For *H. armigera*, they have similar phototactic responses to those of *S. frugiperda*.

### 3.3. Comparison of Phototactic Rates between S. frugiperda and H. armigera

**Figure 4:**
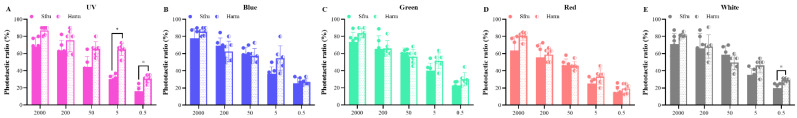
Comparison of phototactic rate between *S. frugiperda* and *H. armigera*. (**A**–**E**), comparison of phototactic rate between *S. frugiperda* and *H. armigera* to different wavelength lights under five light intensities ((**A**) for UV light; (**B**) for blue light; (**C**) for green light; (**D**) for red light; (**E**) for white light). Data are mean ± SE. “*” indicates a significant difference between the two species under the same condition (*p* < 0.05, *t*-test). UV, UV LED; Blue, blue LED; Green, green LED; Red, Red LED; White, white LED.

Phototactic rates of the two moths were compared to different wavelengths and intensities (Figure 4). Under low light intensities of 0.5 and 5 lx to UV light, the phototactic rates of *S. frugiperda* were 16.22% and 30.41%, while they were 28.50% and 64.06% for *H. armigera*. Significant differences were found between the two species under these conditions (Figure 4A, *p* < 0.05). In addition, *S. frugiperda* showed a lower phototactic rate than *H. armigera* (19.64% vs. 29.11%) at the low light intensity of 0.5 lx for white light (Figure 4E, *p* < 0.05).

### 3.4. Phototactic Recovery Rates of Simulated Recovery Experiments for Two Moths

**Figure 5:**
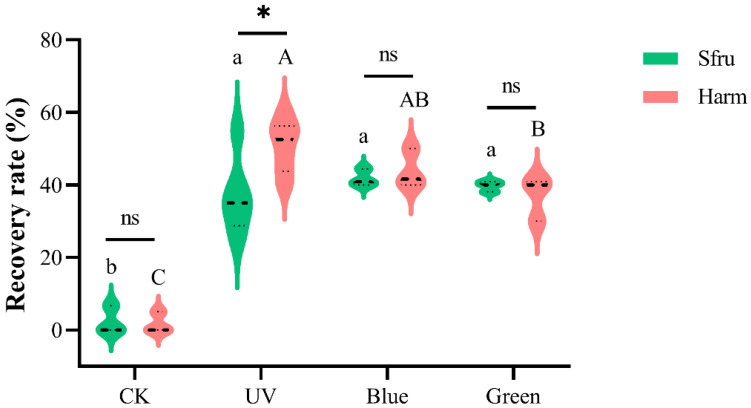
Phototactic recovery rates of *S. frugiperda* and *H. armigera* under indoor simulated conditions. The light intensity is 0.5 lx for three LED lights. Data are mean ± SE. Different letters of the same species indicate significant differences among different treatments (*p* < 0.05, ANOVA). “*” indicates a significant difference between the two samples (*p* < 0.05, *t*-test), while “ns” means not.

Because there were no significant differences in the phototactic rate between sexes, and in order to avoid interference between males and females, only male moths were used in this experiment. As shown in Figure 5, the release recovery rates of the two species to the three lights were significantly higher than those of the controls (*p* < 0.05). For *S. frugiperda*, there were no significant differences in the release recovery rate among the three lights (*p* < 0.05), although the highest value was shown for blue light. In contrast, the release recovery rate of *H. armigera* to UV (50.83%) was markedly higher than that of green light (36.97%) (*p* < 0.05). At the light intensity of 0.5 lx for UV light, the release recovery rate was about 36.67% for *S. frugiperda*, which was significantly lower than that of *H. armigera* (*p* < 0.05). For blue light, no significant difference was found between the two species (41.78% vs. 43.89%, *p* < 0.05). Similar results were obtained for the green light of the two species (39.67% vs. 36.97%, *p* < 0.05).

### 3.5. EC50 and Trapping Distances of Two Moths with Different Light Sources

**Figure 6:**
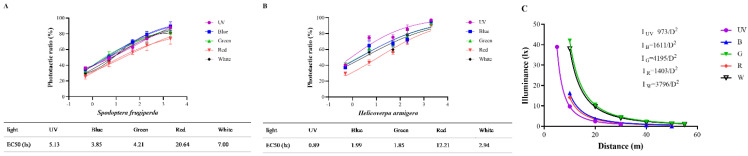
EC50 of *S. frugiperda* and *H. armigera* to different wavelength light sources. The fitting equation was obtained using the software of GraphPad Prism version: 9.3.0, and light intensities were converted by log. EC50 and was calculated by nonlinear regression curve fit and log (agonist) vs. normalized response−variable slope. (**A**) for *S. frugiperda*. (**B**) for *H. armigera*. (**C**) Light intensities of five light sources at different distances were measured and the relationship functions between light intensity and distance of each light source were calculated.

The EC50 of the two species with different light sources is shown in Figure 6. For *S. frugiperda*, the intensities of five light sources at EC50 were 5.13 lx for UV light, 3.85 lx for blue light, 4.21 lx for green light, 20.64 lx for red light, and 7.00 lx for white light. Obviously, the intensity of EC50 for blue and green light was relatively lower for *S. frugiperda*. In the same order, the intensity of EC50 for *H. armigera* to different light sources was 0.89 lx for UV light, 1.99 lx for blue light, 1.85 lx for green light, 12.21 lx for red light, and 2.94 lx for white light. Clearly, the intensity of EC50 for UV light was the lowest for *H. armigera*.

The light intensities of five light sources at different distances were measured (Table S2), and the relationship functions between light intensity and distance of each light source were calculated, as shown in Figure 6C. Combined with the Hill equation and distance function formula, the effective distance could be calculated by phototactic rate and light intensity (Table S3, top and bottom were assigned 100 and 0, respectively). According to the field experiments [32,33,34], the recommended light-trapping distances of *H. armigera* were about 50–100 m. Light-trapping distances calculated by our formula for *H. armigera* (Table 1) were consistent with these field test values, thus indicating that our formula was reliable (Table S3).

Moths of *S. frugiperda* mate at three days old and lay eggs at four days old, so trapping them for three consecutive days before laying eggs is very effective when the adults of *S. frugiperda* appear. The phototactic rates and effective trapping distances of the two moths to blue and UV lights are shown in Table 1. The 50% phototactic rate was instructive, while 10% is another indicator because of the controls (threshold). Among the several light sources, the average phototactic rate of *S. frugiperda* to blue light was the highest. When the phototactic rate was 50% and 10%, the trapping distances of blue light to *S. frugiperda* were 36 and 108 m, respectively. Under the same conditions, the trapping distances of UV light to *H. armigera* were 54 and 163 m, respectively.

## 4. Discussion

Response behaviors and phototactic mechanisms of insects to different light sources are the main objectives of light-trap technology in order to develop pest control technology using new light sources such as LEDs. Wavelength selectivity and high intensity of LEDs enable them to be specifically designed for targeting pests, protecting biodiversity and the environment [26,35]. *S. frugiperda* showed the highest average phototactic rate to blue light among the five different LED lights. The phototactic rates increased with light intensity and were not affected by sex. Under low light intensity, there was a significant difference in the phototactic rate of UV light between *S. frugiperda* and *H. armigera*, further confirmed by indoor simulation experiments. Moreover, the light intensities of *S. frugiperda* under EC50 were more than those of *H. armigera* for different light sources. According to our calculation formula, the trapping distance of blue light to *S. frugiperda* was smaller than that of *H. armigera* to UV light. Therefore, we summarized a proposal of using blue light for a light trap to control *S. frugiperda*, with the maximum distance not exceeding 108 m.

The sensitivity of insects to light is highly wavelength-dependent [22]. The majority of insect species possess classic trichromatic vision, comprising three photoreceptors in their compound eyes, i.e., UV-, blue-, and greenlight sensitive [36,37]; however, visual pigments expressed in photoreceptors have undergone duplication, deletion, and mutation resulting in the diversification of insect light-selection behavior [38,39]. The results of this study showed that *H. armigera* was more sensitive to UV LED, which was consistent with previous reports [5,18]. In contrast, *S. frugiperda* preferred blue or green lights. These results indicated there were differences in the wavelength selectivity between the two moths. Overall, the majority of moths have been reported to be sensitive to UV light [11] but recently many reports have described that some moths prefer other light sources, including purple, blue, and green light [26,40]. In their research, many agricultural insects were more attracted to green or blue LEDs, such as *S. exigua*, and *S. litura* [16]. Similarly, the oriental armyworm, *M. separata*, was sensitive to green light (520 nm) [15]. Even for the same insect, the phototactic behavior results were not consistent. The green LED trap captured more *Plutella xylostella* when compared with UV and other LED lights [41,42], while later it was reported that *P. xylostella* was more sensitive to purple light (400℃405 nm) than other LED lights [43]. In addition, we only studied the phototactic behaviors of *S. frugiperda* against monochromatic light sources. It needs to be further studied whether different effective light sources are synergistic to produce better trapping effects.

Light intensity is another important factor affecting the phototactic behavior of insects. For most insects, they prefer higher light intensity in a certain range at the same wavelength [22,44]. The phototactic rate of the two species increased with the light intensity in our study, which consisted of the phototactic behavior pattern of most insects [22]. In comparison, decreased phototactic responses with increasing light intensities at high intensities for both UV light and blue light were found for a common hyperparasitoid, *Pachyneuron aphidis* (Hymenoptera: Pteromalidae), showing a negative behavior pattern [23]. Moreover, the phototactic response of *Thrips tabaci* was shown to decrease at the middle light intensity, showing a wavy manner [25]. Interestingly, the phototactic behaviors of two weevils were not affected by the light intensity of 200–1000 lx, which was obviously different from the behaviors of other insects [24]. We used lx as the unit of light intensity in our experiments because it was more widely used in actual agricultural productions and was found in many studies (Table S1). However, it is more accurate to use photon units for spectral measurements, like photons/s/cm^2^ and μmol/m^2^/s [45]. The reason is that light-dependent biological processes usually depend on absorbing a photon or not, and photoreceptors are often referred to as photon counters [30]; therefore, this should be paid attention to while doing visual-related research. Sex also has an effect on the phototactic behavior of insects [20]. Most males are captured more by light traps, such as *Ostrinia furnacalis* and *S. exigua*, while females are more often captured only in a few species [19,22,46,47]. For the moth of *Pelosia muscerda*, rates of females and males tending to the light source were equal [20,47]. On the contrary, female moths of *Loxostege sticticalis* (Lepidoptera: Pyralidae) have displayed stronger phototactic responses than males [48]. Field experiments have shown that there are three sex ratios for the two moths, but in our study, no significant difference was found between the sexes. This may be due to the close range of indoor experiments. There needs to be further research on how much the sex ratio in the catches deviates from the natural sex ratio.

When moths respond to a light source, little is known about the trapping distances [49]. The attractiveness of light traps to moths is inversely proportional to distance, which is different among species [50]. It was previously thought that the distance of the light trap was far away and ranged from 50 to 500 m depending on the species when using a certain type of light trap [49,51,52,53,54]. Nevertheless, the actual trapping distances were much nearer than speculated. Through recapture experiments, distances of moths’ responses to light were surprisingly short, only a few meters, and generally no more than 40 m [49,53,55]. Based on the investigation of pest occurrence and crop damage rate, the recommended light trapping distances for *H. armigera* were about 50–100 m [32,33,34]. The trapping distances calculated by our formula for *H. armigera* were consistent with these field test values, which showed that our formula was reliable. Meanwhile, the relationship between different phototactic rates and trapping distances can be obtained through our formula (Table S3). According to actual needs, the distance of light traps can be optimized and adjusted. For example, when pest control is carried out with a large occurrence of *S. frugiperda*, the cumulative phototactic rate can reach 87.50% and the trapping distance is about 36 m, taking 50% of the phototactic rate as the index. However, in pest monitoring, the trapping distance can be set to as far as 108 m with the threshold value of 10% as the indicator corresponding to our results of the controls that are in the dark. *S. frugiperda* had the highest average phototactic rate to blue light than other LED lights. Considering environmental protection and energy requirements, the blue LED has higher illumination intensity and longer effective trapping distance than UV LED under the same wattage, so the blue LED light can be used as an effective light source. Some moths were sensitive to blue light and were captured by the blue LED trap [18,20,40]. In sum, the distances of moths to light sources are varied and could not be a constant distance, which is a basis for the field application of this technology for different target insects [50].

## 5. Conclusions

In summary, *S. frugiperda* had the highest average phototactic rate for blue light than other LED lights, compared to *H. armigera*, which showed a maximum preference for UV light. The phototactic rates of two moths increased with light intensity and were not affected by sex. Meanwhile, the phototactic rates of *S. frugiperda* were significantly lower than those of *H. armigera* at a low light intensity of UV light, further confirmed by the indoor simulation experiment and EC50. According to the obtained parameters, the trapping distance of *S. frugiperda* to blue light was smaller than that of *H. armigera* to UV light. Therefore, we summarized a proposal of using blue light for light traps to control *S. frugiperda*, with a maximum distance of no more than 108 m.

## Figures and Tables

**Table 1:** Phototactic rates and trapping distances of two moths to different lights.

Species	Light Source	P1 (%)	P3 (%)	Distance (m)
*S. frugiperda*	blue	50	87.5	36
		10 *	27.1	108
*H. armigera*	UV	50	87.5	54
		10 *	27.1	163

P_1_, phototactic rate of one night; P_3_, phototactic rate for three consecutive days. * 10% was the threshold of the controls in the dark.

## Data Availability

The data presented in this study are available in the article.

## Supplementary Materials

The following supporting information can be downloaded at: https://www.mdpi.com/article/10.3390/insects13100917/s1: Figure S1: The spectrum of LEDs used for the experiments; Figure S2: Phototactic rates of *Spodoptera frugiperda* and *Helicoverpa armigera* under negative control without light; Table S1: Description of illumination intensity (lx) of different light sources [56,57,58,59,60,61]; Table S2: Distances from light source and detected light intensities; Table S3: Function between trapping distance and phototactic rate for *S. frugiperda* and *H. armigera*.
