# Supplementary material for: Comparison of Phototactic Behavior between Two Migratory Pests, Helicoverpa armigera and Spodoptera frugiperda
Source: Insects. 2022 Oct 9;13(10):917. doi: 10.3390/insects13100917 (PMC9603935; doi:10.3390/insects13100917)
Supplement: Supplementary file 1 [file insects-13-00917-s001.zip › Tables S1-S3.pdf]

Table S1. Description of illumination intensity (Lx) of different light sources*

Year	Journal	Description of light intensity
2022	*Agronomy*	The light intensity of the experiment was set as 50 lux for the phototactic reaction experiment of different spectra.
2021	*Int. J. Agric. & Biol. Eng.*	Experimental illumination of the UV, violet, orange, and green light sources, as calibrated by an illuminance meter (model ST-80C) by adjusting the PWM modulator, was 1000 lx (UV, violet) and 10 000 lx (orange, green). Thus, when light spectra were different, by increasing the intensity of light, the same reaction effect of locusts' visual system could be obtained.
2020	*Coleopts. Bull.*	The experimental treatments included 15 different narrow bands of monochromatic light obtained by various filters. The light intensity of the different filters was standardized to a constant 10 lux by adjusting the slide rheostat.
2018	*Biocontrol Sci. Techn.*	Funnel traps were made with LED lights at eight different wavelengths. ……Light intensity was measured at a distance of more than 10 cm above the lamp and was adjusted to 1000 lux using a sliding rheostat.
2018	*J. Asia-Pac. Entomol.*	Fig. 2. Phototactic responses of *M. separata* adults to LED lights of several wavelengths. The experiment was executed under 200 lx (luminance intensity) and 40 min (exposure time) conditions for each group.
2017	*Int. J. Agric. & Biol. Eng.*	LED spectral stimuli were provided with peak wavelengths at 400 nm, 450 nm, 520 nm and 610 nm. The stimuli were calibrated by an illuminance meter (model-ST-80C) and controlled by a PWM modulator to a set 100 lx.
2017	*Appl. Biol. Chem.*	*Bemisia tabaci* and *Trialeurodes vaporariorum* showed a significantly more favorable response to the green (520 nm) and blue (470 nm) LEDs at a luminance intensity of 40 lx and exposure time of 90 min than to red (625 nm) and yellow (590 nm) LEDs.
2016	*J. Appl. Biol. Chem.*	The attractive rates of the *T. castaneum* adults to the LEDs of seven different wavelengths (UV, blue, green, yellow, red, white, and IR) and five light-exposure times (12, 24, 36, 48, and 60 h) were examined at optimal luminous intensity (50 lx).
2016	*J. Insect Behav.*	We used nine different optical filters with center wavelengths of monochromatic light ranging from 340 nm to 689 nm with ~40-nm steps. The intensity of monochromatic light was kept at 10 lx.
2016	*Biocontrol Sci. Techn.*	The light intensity for the different filters was measured by an illuminometer (SMART SENDOR AR823) and was standardised at a constant 10 lux by adjusting the slide rheostat.

* Articles were collected since 2016.

References:

1. Shang, X.K.; Pan, X.H.; Liu, W.; Wei, J.L.; Huang, C.H.; Goebel, F.; Nikpay, A. Effect of spectral sensitivity and light intensity response on the phototactic behavior of *Exolontha castanea* Chang

(Coleoptera: Melolonthidae), a pest of sugarcane in China. *Agronomy* 2022, 12, 1-13, doi:10.3390/agronomy12020481.

2. Liu, Q.; Jiang, Y.; Miao, J.; Gong, Z.; Li, T.; Duan, Y.; Wu, Y. Photoreceptive reaction spectrum effect and phototactic activity intensity of locusts' visual display characteristics stimulated by spectral light. *Int. J. Agric. & Biol. Eng.* 2021, 14, 19-25, doi:10.25165/j.ijabe.20211402.4758.
3. Chen, Z.; Li, H.-M.; Zhou, C.-L. Phototactic Behavior of *Harmonia axyridis* (Pallas) (Coleoptera: Coccinellidae). *Coleopts. Bull.* 2020, 74, doi:10.1649/0010-065x-74.3.513.
4. Wen, C.; Ji, Y.C.; Zhang, G.Y.; Tan, S.B.; Wen, J.B. Phototactic behaviour of *Eucryptorrhynchus scrobiculatus* and *E. brandti* (Coleoptera: Curculionidae) adults. *Biocontrol Sci. Techn.* 2018, 28, 544-561, doi:10.1080/09583157.2018.1464124.
5. Kim, K.N.; Song, H.S.; Li, C.S.; Huang, Q.Y.; Lei, C.H. Effect of several factors on the phototactic response of the oriental armyworm, *Mythimna separata* (Lepidoptera: Noctuidae). *J. Asia-Pac. Entomol.* 2018, 21, 952-957, doi:10.1016/j.aspen.2018.07.010.
6. Liu, Q.H.; Xin, Z.; Zhou, Q. Visual reaction effects induced and stimulated by different lights on phototactic bio-behaviors in *Locusta migratoria manilensis*. *Int. J. Agric. & Biol. Eng.* 2017, 10, 173-181, doi:10.25165/j.ijabe.20171004.2357.
7. Park, J.H.; Lee, H.S. Phototactic behavioral response of agricultural insects and stored-product insects to light-emitting diodes (LEDs). *Appl. Biol. Chem.* 2017, 60, 137-144, doi:10.1007/s13765-017-0263-2.
8. Song, J.; Jeong, E.-Y.; Lee, H.-S. Phototactic behavior 9: phototactic behavioral response of *Tribolium castaneum* (Herbst) to light-emitting diodes of seven different wavelengths. *J. Appl. Biol. Chem.* 2016, 59, 99-102, doi:10.3839/jabc.2016.018.
9. Luo, C.-W.; Chen, Y. Phototactic Behavior of *Scleroderma guani* (Hymenoptera: Bethylidae) - Parasitoid of *Pissodes punctatus* (Coleoptera: Curculionidae). *J. Insect Behav.* 2016, 29, 605-614, doi:10.1007/s10905-016-9584-y.
10. Chen, Z.; Xu, R.; Kuang, R.p.; Sun, R. Phototactic behaviour of the parasitoid *Encarsia Formosa* (Hymenoptera: Aphelinidae). *Biocontrol Sci. Techn.* 2016, 26, 250-262, doi:10.1080/09583157.2015.1099071.

Table S2. Distances from light source and detected light intensities

Distance (m)	Light intensity (Lx)				
	UV	Blue	Green	Red	White
10	9.7	16.3	42.0	14.2	38.0
20	2.4	3.7	10.6	3.3	9.7
30	1.1	1.3	4.4	1.0	4.0
40	0.6	0.5	2.2	0.3	1.8
50	0.4	0.1	1.3	0.1	1.1

Table S3. Function between trapping distance and phototactic rate for *S. frugiperda* and *H. armigera*

Species	Light	Function
S. frugiperda	blue	$D=\sqrt{(100-P)*\frac{1611}{P*0.32*3.85}}$
H. armigera	UV	$D=\sqrt{(100-P)*\frac{973}{P*0.37*0.89}}$

P, phototactic rate of one night (%); D, trapping distance.
