# Supplementary figures and images for: Comparison of Phototactic Behavior between Two Migratory Pests, Helicoverpa armigera and Spodoptera frugiperda
Source: Insects. 2022 Oct 9;13(10):917. doi: 10.3390/insects13100917 (PMC9603935; doi:10.3390/insects13100917)

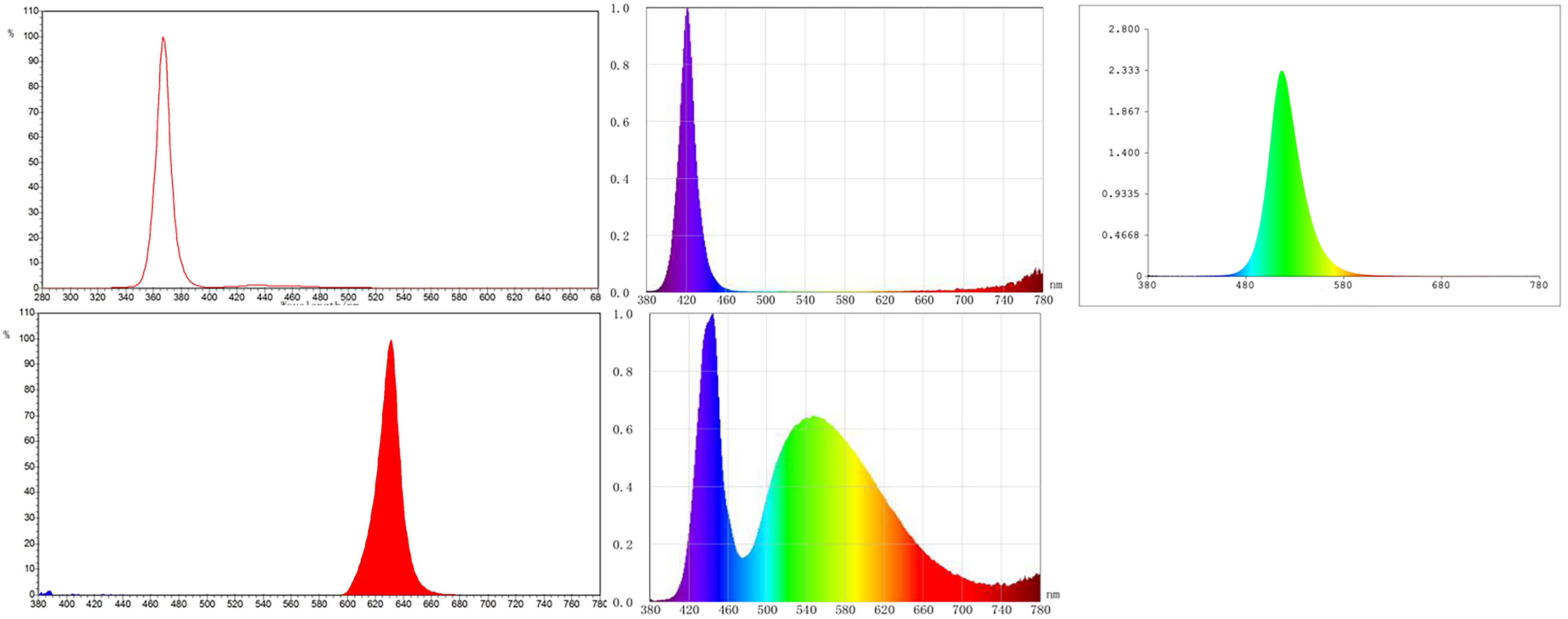

Supplement: Supplementary file 1 [file insects-13-00917-s001.zip › Figure S1.tif]

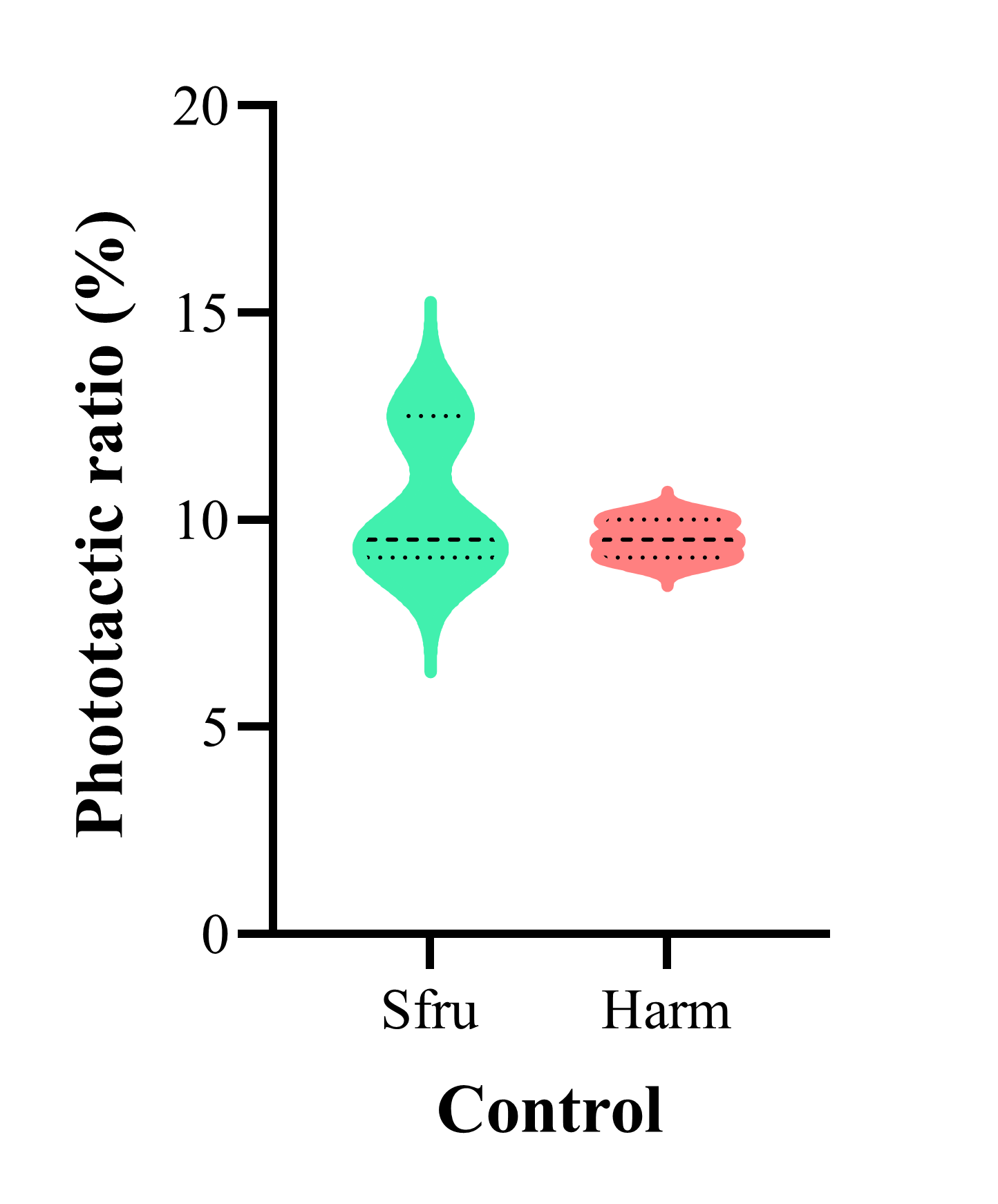

Supplement: Supplementary file 1 [file insects-13-00917-s001.zip › Figure S2.tif]
